# Design, Synthesis, and Bioactivity Study of Novel Tryptophan Derivatives Containing Azepine and Acylhydrazone Moieties

**DOI:** 10.3390/molecules27196700

**Published:** 2022-10-08

**Authors:** Jingjing Zhang, Rongxin Yang, Lili Li, Jianhua Liu, Yuxiu Liu, Hongjian Song, Qingmin Wang

**Affiliations:** 1 College of Basic Science, Tianjin Agricultural University, Tianjin 300384, China; 2 State Key Laboratory of Elemento-Organic Chemistry, College of Chemistry, Frontiers Science Center for New Organic Matter, Nankai University, Tianjin 300071, China

**Keywords:** tryptophan derivatives, azepino [4,5-*b*] indole, acylhydrazone, antiviral activity, larvicidal activity, antifungal activity

## Abstract

Based on the scaffolds widely used in drug design, a series of novel tryptophan derivatives containing azepine and acylhydrazone moieties have been designed, synthesized, characterized, and evaluated for their biological activities. The bioassay results showed that the target compounds possessed moderate to good antiviral activities against the tobacco mosaic virus (TMV), among which compounds **5c**, **6a**, **6h**, **6t**, **6v**, and **6y** exhibited higher inactivation, curative, and protection activities in vivo than that of ribavirin (40 ± 1, 37 ± 1, 39 ± 2% at 500 mg/L). Especially, **6y** showed comparable activities to that of ningnanmycin (57 ± 2, 55 ± 3, 58 ± 1% at 500 mg/L). Meanwhile, we were pleased to find that almost all these derivatives showed good larvicidal activities against *Plutella xylostella*. Meanwhile, these derivatives also showed a broad spectrum of fungicidal activities.

## 1. Introduction

Natural products are secondary metabolites retained by natural organisms through long-term evolution and natural selection [1]. Because natural products often have the characteristics of chemical structure and biological activity diversity, they have great value in the development and utilization of drugs [2,3,4,5].

L-Tryptophan is not only an essential amino acid that constitutes the proteins of living substance, but is also a very important structural unit in the biosynthesis of natural products [6]. Due to its multiple functional groups, such as carboxyl, amino, and indole groups, tryptophan can be modified to form a variety of different structural units. Furthermore, some tryptophan derivatives also have great value in the fields of biology and medicine [7]. Although there is a lot of research on the biological activities of tryptophan, there are few studies focused on its application in the field of pesticides. In our previous work, we found, for the first time, that tryptophan exhibits antiplant virus activity [8]. Therefore, it can be used as an antiviral lead for structural optimization.

**Figure 1:**
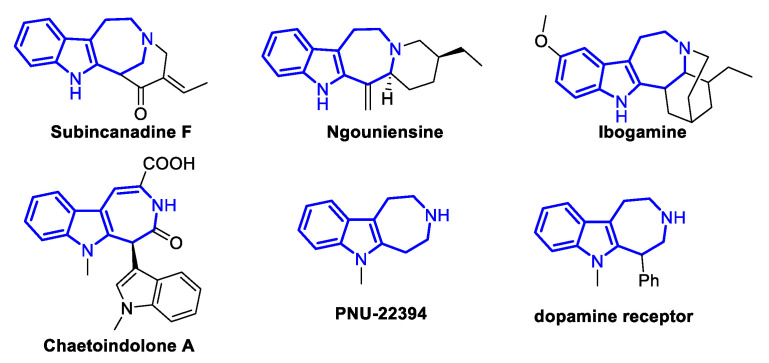
Natural products and pharmaceuticals containing azepine structures.

Azepines are important structural motifs that widely exist in natural products and pharmaceuticals [9,10,11,12] (Figure 1). Due to their special structure type and electron cloud distribution, azepines have been widely used in the design of antitumor [13,14,15], antihypertension [16], anti-inflammatory [17], antibacterial, and antifungal [18] drugs.

Acylhydrazones are products obtained by combining hydrazides with ketones or aldehydes. The bioactive acylhydrazone core has been one of the most ubiquitous functional groups in medicinal chemistry, and it has been identified in a huge number of lead compounds that act on various types of molecular targets [19,20,21,22,23]. In our previous work, it was found that the introduction of acylhydrazone fragments was very beneficial to improve the antiplant virus of the compound [24,25,26].

**Figure 2:**
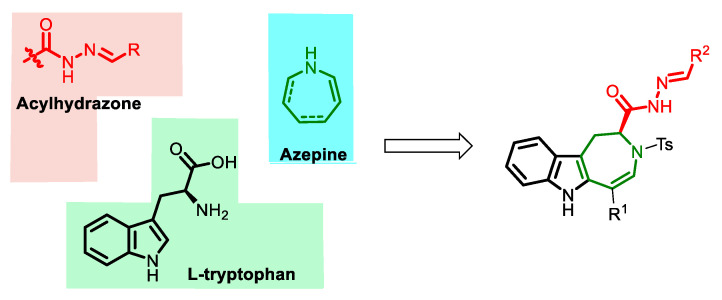
Design of target compounds.

In this work, to improve the antivirus activity of tryptophan, we designed and synthesized a series of novel tryptophan derivatives containing azepine and acylhydrazone moieties and first evaluated their biological activities (Figure 2). In addition, the larvicidal and fungicidal activities of the newly synthesized tryptophan derivatives were also studied to expand their potential agricultural applications.

## 2. Results

### 2.1. Chemistry

**Scheme 1:**
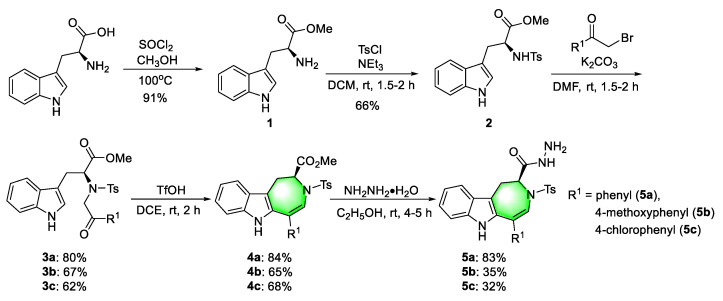
Synthesis of **5a**−**5c**.

**Scheme 2:**
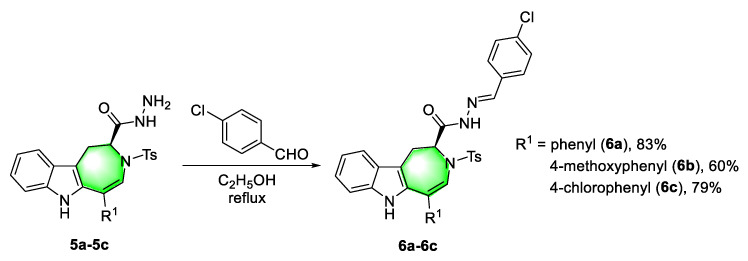
Synthesis of **6a**−**6c**.

**Scheme 3:**
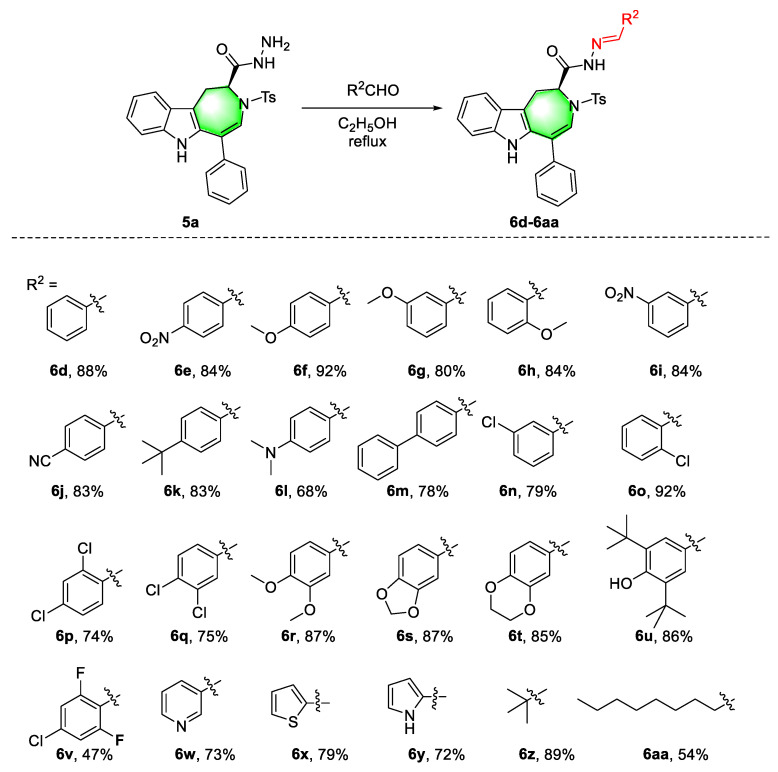
Synthesis of **6d**−**6aa**.

Tryptophan underwent esterification, sulfonylation, nucleophilic substitution, cyclization under acidic conditions, and finally, hydrazinolysis of esters (**4a**–**4c**) to obtain hydrazide compounds **5a**–**5c** (Scheme 1). Hydrazide compounds **5a**–**5c** reacted with 4-chlorobenzaldehyde through a condensation reaction to give **6a**–**6c** (Scheme 2). Additionally, **6a**–**6aa** was synthesized by the condensation reaction between **5a** and corresponding aldehydes (Scheme 3).

### 2.2. Antiviral Activities

Using the commercial plant viricides ningnanmycin and ribavirin as controls, we first evaluated the inactivation effect of compounds **5a**–**5c** and **6a**–**6aa** against TMV in vivo at 500 mg/L, and then the curative and protective modes of antiviral activity were tested at both 500 and 100 mg/L for these compounds, with more than a 40% in vivo inactivation effect at 500 mg/L (Table 1). The bioassay results showed that most of these compounds were active against TMV, and some of these compounds exhibited better anti-TMV activities than ribavirin, such as hydrazide compounds (**5b** and **5c**) and acylhydrazone compounds (**6a**, **6e**, **6g**, **6h**, **6m**, **6n**, **6t**, **6v**, **6y)**. In particular, **5c**, **6a**, **6h**, **6t**, **6v**, and **6y** exhibited comparable activity with ningnanmycin. 

To compare the structure–activity relationship between hydrazide and acyhydrazone compounds, we studied the anti-TMV activity of compounds **5a–5c** and **6a–6c**. The bioassay results showed that these two types of compounds showed different structure–activity relationships: For the hydrazide compounds **5a–5c**, the effect of R^1^ on anti-TMV activity was shown as 4-chlorophenyl (**5c**) > 4-methoxyphenyl (**5b**) > phenyl (**5a**). In contrast, after the introduction of the acylhydrazone moiety, only the anti-TMV activity of **6a** was improved, and the antiviral activities of the three compounds decreased in the order **6a** > **6b** ≈ **6c**.

For these acyhydrazone compounds, when R^1^ was phenyl, the types of R^2^ and the substituents on it (R^2^) had important effects on the anti-TMV activity. When R^2^ was phenyl, the types, positions, and number of the substituents on the benzene ring had a significant effect on the anti-TMV activity of these compounds (**6d–6z**). Furthermore, **6a** (R^2^ = 4-chlorophenyl), **6h** (R^2^ = 2-methoxyphenyl), **6t** (R^2^ = 2,3-dihydrobenzo[b][1,4]dioxin-6-yl), and **6v** (R^2^ = 4-bromo-2,6-difluorobenzyl) exhibited higher anti-TMV activities than other compounds. The positions of these substituents on the benzene ring also affected the anti-TMV activities; moreover, the influence of the position of these substituents with different electrical properties on the activity was also different. For the electron-withdrawing group (such as chloro) on the benzene ring, the order of their activity level was para (**6a**) > meta (**6n**) > ortho (**6o**). However, for the electron-donating group (such as methoxy) on the benzene ring, the order of their activity level was ortho (**6h**) > meta (**6g**) > para (**6f**). In addition, the number of substituents on benzene rings also influenced the anti-TMV activity, whether it was for the electron-withdrawing group or the electron-donating group; the more the number of substituents on the benzene ring, the lower the anti-TMV activity of these compounds. For example, compounds **6p**, **6q**, and **6r** showed lower anti-TMV activity than monosubstituted compounds. Noteworthily, **6t** (R^2^ = 2,3-dihydrobenzo[b][1,4]dioxin-6-yl) showed higher activity than **6s** (R^2^ = benzo[*d*][1,3]dioxo-5yl) and **6r** (R^2^ = 3,4-dimethoxyphenyl).

When R^2^ was aromatic heterocyclic (**6w–6y**), these compounds exhibited the following structure–activity relationship: **6y** (R^2^ = 1*H*-pyrrol-2yl) > **6x** (R^2^ = thiophene-2-yl) > **6w** (R^2^ = pyridin-3-yl).

When R^2^ was the aliphatic substituent (**6z** and **6aa**), the anti-TMV activities of these two compounds were lower than that of ribavirin. This suggested that aliphatic substituents were unfavorable for improving the anti-TMV activity of these compounds.

### 2.3. Larvicidal Activity

In general, almost all the compounds showed larvicidal activity against *Plutella xylostella*, and especially, **6k**, **6m**, and **6z** showed >80% larvicidal activities at 100 mg/L (Table 2). By comparing the larvicidal activities of hydrazide compounds **5a–5c** and acylhydrazones compounds **6a–6c**, the introduction of benzylidene was detrimental to the larvicidal activity. For these acyhydrazone compounds, the introduction of substituents on benzylidene had a significant effect on the larvicidal activity, and the introduction of lipophilic substituents was beneficial to the larvicidal activity, such as tert-butyl (**6k**) and phenyl (**6m**). The introduction of the heteroaromatic ring (R^2^) had no obvious improvement in larvicidal activity (**6w–6y**). For aliphatic substituents (**6z** and **6aa**), the introduction of tert-butyl (**6z**) could improve the larvicidal activity of the compound (LC_50_ was 21.2 mg/L, Table 3). The above bioassay results showed that the introduction of lipophilic substituents was beneficial to larvicidal activity, which provided useful information for subsequent structural optimization.

### 2.4. Fungicidal Activities

Finally, we investigated the antiphytopathogenic activity of these compounds (Table 4). The fungicidal activities of these compounds against 14 kinds of phytopathogenic fungi were evaluated using the mycelial growth method. Most of these compounds showed fungicidal activities against 14 kinds of plant pathogens at 50 mg/L. These compounds showed selectivity against these fungi, and most compounds exhibited >60% fungicidal activity against *Fusarium moniliforme* at 50 mg/L. Some compounds exhibited broad-spectrum fungicidal activities; compounds **5a** and **5c** showed > 60% fungicidal activity against five kinds of plant pathogens at 50 mg/L, and compounds **6r** and **6aa** showed > 60% fungicidal activity against six kinds of plant pathogens at the same concentration. Some compounds showed excellent fungicidal activity against a certain species of fungus selectively, such as **5a**, **6d**, **6m**, **6t**, and **6w** that showed 90 ± 1%, 92 ± 1%, 100%, 98 ± 1%, and 90 ± 0% inhibition rates against *Rhizoctonia cerealis*, respectively. In the meantime, **6t** showed 94 ± 1% and 100% inhibition rates against *Cercospora arachidicola* Hori and *Fusarium moniliforme*, respectively.

## 3. Materials and Methods

### 3.1. Materials

^1^H, ^13^C nuclear magnetic resonance (NMR) spectra were obtained at 400 MHz and 100 MHz using a Bruker AC-P 400. Chemical shift values (δ) were given in parts per million (ppm) and were downfield from internal tetramethylsilane. High-resolution mass spectra (HRMS) data were obtained on an FTICR-MS instrument (Ionspec 7.0 T). The melting points were determined on an X-4 binocular microscope melting point apparatus and were uncorrected. Reaction progress was monitored via thin-layer chromatography on silica gel GF-254 with detection by UV.

Ribavirin (Topscience Co. Hongkong, China), ningnanmycin (Alta Scientific Co. Tianjin, China), chlorothalonil (Bailing Agrochemical Co. Jiangyin, China), rotenone (Accela ChemBio Inc. Shanghai, China), and other reagents were purchased from commercial sources and were used as received.

### 3.2. General Synthesis

The synthetic routes of target compounds **5a**−**5c**, **6a**−**6aa** are depicted in Scheme 1, Scheme 2 and Scheme 3 [27]. The spectra of target compounds **5a**−**5c**, **6a**−**6aa** are depicted in the Supplemetary Materials, Section S1: Copies of NMR spectra (Figures S1–S76).

#### 3.2.1. Synthesis of (S)-methyl 2-amino-3-(1H-indol-3-yl)propanoate (1)

To a solution of L-tryptophan (10 g, 48.97 mmol) in anhydrous methanol (150 mL), SOCl_2_ (10 mL) was slowly added dropwise and then heated at 100 °C. When the reaction was complete, as indicated by thin-layer chromatography (5 h), the reaction mixture was cooled to room temperature, and then, the mixture was concentrated in vacuo and washed with the anhydrous Na_2_CO_3_ saturated solution, extracted with ethyl acetate (50 mL × 3), and the combined organic phases were washed with brine, dried over Na_2_SO_4_, and filtered; the filtrate was evaporated under reduced pressure to give a brown solid (9.71 g, 91%, mp 90–91 °C). ^1^H NMR (400 MHz, CDCl_3_) δ 8.35 (s, 1H), 7.61 (d, J = 7.6 Hz, 1H), 7.33 (d, J = 8.0 Hz, 1H), 7.19 (t, J = 7.6 Hz, 1H), 7.12 (t, J = 7.6 Hz, 1H), 7.02 (d, J = 2.0 Hz, 1H), 3.84 (dd, J = 7.6, 4.8 Hz, 1H), 3.71 (s, 3H), 3.28 (dd, J = 14.4, 4.8 Hz, 1H), 3.05 (dd, J = 14.4, 8.0 Hz, 1H), 1.64 (s, 2H). ^13^C NMR (100 MHz, CDCl_3_) δ 175.8, 136.3, 127.5, 123.0, 122.2, 119.5, 118.8, 111.3, 111.0, 55.0, 52.1, 30.8.

#### 3.2.2. Synthesis of (S)-methyl 3-(1H-indol-3-yl)-2-(4-methylphenylsulfonamido)propanoate (2)

To a mixture of **1** (8.63 g, 39.5 mmol) and NEt_3_ (11 mL, 79 mmol) in anhydrous dichloromethane (120 mL), para toluenesulfonyl chloride (7.69 g, 59.3 mmol) was slowly added under an ice bath condition, and the mixture was continuously stirred for 2 h at room temperature. When TLC indicated that the reaction was complete, water was added to quench the reaction, and it was extracted by ethyl acetate, washed with brine, dried with anhydrous Na_2_SO_4_, and concentrated in vacuo. The product was purified via chromatography on a column of silica gel (dichloromethane: methanol = 20:1) to offer a white solid (9.63 g, 66%), mp 100–101 °C. ^1^H NMR (400 MHz, CDCl_3_) δ 8.09 (s, 1H), 7.60 (d, J = 8.4 Hz, 2H), 7.43 (d, J = 8.0 Hz, 1H), 7.32 (d, J = 8.0 Hz, 1H), 7.22−7.14 (m, 3H), 7.06 (t, J = 7.2 Hz, 1H), 7.02 (d, J = 2.4 Hz, 1H), 5.12 (d, J = 9.2 Hz, 1H), 4.29−4.17 (m, 1H), 3.43 (s, 3H), 3.23 (d, J = 5.6 Hz, 2H), 2.37 (s, 3H). ^13^C NMR (100 MHz, CDCl_3_) δ 171.8, 143.5, 136.4, 136.1, 129.5, 127.1, 123.6, 122.1, 119.6, 118.4, 111.4, 108.8, 56.0, 52.5, 29.2, 21.5.

#### 3.2.3. General Procedures for the Preparation of Compounds 3a–3c

A mixture of compound **2** (2 mmol) and K_2_CO_3_ (1.5 equiv) in DMF (10 mL) was stirred at 0^o^C for 30 min; then, 2-bromoacetophenones (1.2 equiv) were added and the mixture was warmed naturally to room temperature and stirred till the complete consumption of the starting materials. When TLC indicated that the reaction was complete, the reaction was quenched with ice-cold water and extracted with ethyl acetate. The combined organic layer was washed with ice-cold water and saturated with aqueous ammonium chloride, dried with anhydrous Na_2_SO_4_, concentrated in vacuo, and then purified by chromatography on a column of silica gel to offer **3a**–**3c**.

(S)-3-methyl (1H-indol-3-yl)-2-(4-methyl-N-(2-oxo-2-phenylethyl)phenylsulfon-amido)propanoate (**3a**):

Yellow solid, yield 80%, mp 118–120 °C. ^1^H NMR (400 MHz, CDCl_3_) δ 7.99−7.93 (m, 3H), 7.84 (d, J = 8.4 Hz, 2H), 7.60 (t, J = 7.6 Hz, 1H), 7.50 (t, J = 7.6 Hz, 2H), 7.41 (d, J = 8.0 Hz, 1H), 7.31 (d, J = 8.0 Hz, 1H), 7.24 (d, J = 8.0 Hz, 2H), 7.17 (t, J = 7.2 Hz, 1H), 7.10−7.05 (m, 1H), 6.95 (d, J = 2.4 Hz, 1H), 5.11 (d, J = 1.6 Hz, 2H), 4.65 (dd, J = 9.7, 5.3 Hz, 1H), 3.42 (s, 3H), 3.24 (dd, J = 14.0, 9.6 Hz, 1H), 3.14 (dd, J = 14.0, 5.2 Hz, 1H), 2.41 (s, 3H). ^13^C NMR (100 MHz, DMSO) δ 189.1, 166.1, 138.5, 131.3, 130.9, 129.8, 128.4, 124.1, 123.6, 122.9, 122.8, 121.7, 118.1, 116.8, 114.3, 113.1, 106.1, 104.3, 54.0, 46.8, 44.8, 21.6, 16.4.

(S)-methyl 3-(1H-indol-3-yl)-2-(N-(2-(4-methoxyphenyl)-2-oxoethyl)-4-methylphenylsulfonamido)propanoate (**3b**):

Yellow solid, yield 67%, mp 100–102 °C. ^1^H NMR (400 MHz, CDCl_3_) δ 8.12 (s, 1H), 7.94 (d, J = 8.0 Hz, 2H), 7.83 (d, J = 7.6 Hz, 2H), 7.39 (d, J = 7.6 Hz, 1H), 7.28 (d, J = 8.0 Hz, 1H), 7.21 (d, J = 8.0 Hz, 2H), 7.14 (t, J = 7.6 Hz, 1H), 7.05 (t, J = 7.6 Hz, 1H), 6.96−6.90 (m, 3H), 5.06 (s, 2H), 4.64 (dd, J = 9.6, 5.2 Hz, 1H), 3.87 (s, 3H), 3.39 (s, 3H), 3.23 (dd, J = 14.0, 9.6 Hz, 1H), 3.13 (dd, J = 14.0, 5.2 Hz, 1H), 2.38 (s, 3H). ^13^C NMR (100 MHz, CDCl_3_) δ 192.7, 171.3, 163.9, 143.6, 136.6, 136.1, 130.3, 129.3, 128.1, 128.1, 127.0, 123.4, 122.0, 119.5, 118.4, 114.0, 111.2, 109.8, 59.3, 55.6, 52.0, 49.7, 26.8, 21.6.

(S)-methyl2-(N-(2-(4-chlorophenyl)-2-oxoethyl)-4-methylphenylsulfonamido)-3-(1H-indol-3-yl)propanoate (**3c**):

White solid, yield 62%, mp 80–81 °C. ^1^H NMR (400 MHz, CDCl_3_) δ 7.96 (s, 1H), 7.90 (d, J = 8.8 Hz, 2H), 7.82 (d, J = 8.4 Hz, 2H), 7.46 (d, J = 8.8 Hz, 2H), 7.40 (d, J = 8.0 Hz, 1H), 7.31 (d, J = 8.4 Hz, 1H), 7.24 (d, J = 8.0 Hz, 2H), 7.17 (t, J = 7.2 Hz, 1H), 7.08 (t, J = 7.2 Hz, 1H), 6.95 (d, J = 2.4 Hz, 1H), 5.05 (s, 2H), 4.64 (dd, J = 9.6, 5.6 Hz, 1H), 3.42 (s, 3H), 3.23 (dd, J = 14.0, 9.6 Hz, 1H), 3.13 (dd, J = 14.0, 5.2 Hz, 1H), 2.41 (s, 3H). ^13^C NMR (100 MHz, CDCl_3_) δ 193.2, 171.3, 143.7, 140.1, 136.4, 136.1, 133.4, 129.4, 129.4, 129.1, 128.1, 127.0, 123.2, 122.1, 119.6, 118.4, 111.2, 109.7, 59.1, 52.1, 49.9, 26.8, 21.6.

#### 3.2.4. General Procedures for the Preparation of Compounds **4a**–**4c**

A solution of **3** (0.5 mmol) in 1,2-dichloroethane was added with trifluoromethanesulfonic acid (0.2 equiv) and stirred at room temperature. When TLC indicated that the reaction was complete, water was added, and it was extracted with dichloromethane; then, the combined organic phases were washed with brine, dried over anhydrous Na_2_SO_4_, concentrated in vacuo, and finally, purified by chromatography on a column of silica gel (petroleum ether: ethyl acetate = 5:1) to obtain the desired product **4a**–**4c**.

(S)-methyl 5-phenyl-3-tosyl-1,2,3,6-tetrahydroazepino [4,5-b]indole-2-carboxylate (**4a**):

Yellow solid, yield 84%, mp 88–90 °C. ^1^H NMR (400 MHz, CDCl_3_) δ 7.75 (d, J = 8.4 Hz, 2H), 7.52 (s, 1H), 7.48−7.38 (m, 6H), 7.32 (d, J = 8.0 Hz, 2H), 7.15−7.03 (m, 3H), 6.98 (d, J = 0.4 Hz, 1H), 5.57 (t, J = 3.6 Hz, 1H), 3.92 (dd, J = 16.0, 4.8 Hz, 1H), 3.28 (s, 3H), 2.79 (dd, J = 16.0, 3.2 Hz, 1H), 2.42 (s, 3H). ^13^C NMR (100 MHz, CDCl_3_) δ 168.0, 144.4, 138.7, 135.6, 135.1, 131.2, 130.0, 129.7, 129.0, 128.4, 128.0, 127.3, 124.4, 122.4, 119.7, 117.7, 116.5, 110.7, 110.6, 57.6, 52.2, 28.3, 21.7.

(S)-methyl 5-(4-methoxyphenyl)-3-tosyl-1,2,3,6-tetrahydroazepino [4,5-b]indole-2-carboxylate (**4b**):

Yellow solid, yield 65%, mp 95–97 °C. ^1^H NMR (400 MHz, CDCl_3_) δ 7.74 (d, J = 8.0 Hz, 2H), 7.56 (s, 1H), 7.46 (d, J = 7.6 Hz, 1H), 7.31 (t, J = 8.4 Hz, 4H), 7.14−7.03 (m, 3H), 6.96 (d, J = 8.4 Hz, 2H), 6.93 (s, 1H), 5.56 (s, 1H), 3.90 (dd, J = 16.0, 4.8 Hz, 1H), 3.86 (s, 3H), 3.27 (s, 3H), 2.77 (dd, J = 16.0, 2.4 Hz, 1H), 2.40 (s, 3H). ^13^C NMR (100 MHz, CDCl_3_) δ 168.1, 159.5, 144.3, 135.7, 135.1, 131.6, 130.9, 130.9, 129.9, 128.5, 127.3, 124.0, 122.3, 119.7, 117.7, 116.3, 114.3, 110.7, 110.5, 57.7, 55.4, 52.2, 28.2, 21.6.

(S)-methyl 5-(4-chlorophenyl)-3-tosyl-1,2,3,6-tetrahydroazepino [4,5-b]indole-2-carboxylate (**4c**):

Yellow solid, yield 68%, mp 94–96 °C. ^1^H NMR (400 MHz, CDCl_3_) δ 7.73 (d, J = 8.4 Hz, 2H), 7.52 (s, 1H), 7.46 (d, J = 7.2 Hz, 1H), 7.44−7.38 (m, 2H), 7.37−7.29 (m, 4H), 7.16−7.04 (m, 3H), 6.97 (s, 1H), 5.59−5.51 (m, 1H), 3.91 (dd, J = 16.0, 5.2 Hz, 1H), 3.24 (s, 3H), 2.76 (dd, J = 16.0, 2.8 Hz, 1H), 2.41 (s, 3H). ^13^C NMR (100 MHz, CDCl_3_) δ 167.9, 144.6, 137.2, 135.4, 135.2, 134.0, 131.0, 130.9, 130.0, 129.2, 128.4, 127.3, 124.5, 122.5, 119.9, 117.7, 115.0, 110.9, 110.8, 57.7, 52.2, 28.3, 21.7.

#### 3.2.5. General Procedures for the Preparation of Compounds **5a**–**5c**

Ta a solution of **4** (1 equiv) in ethanol (20 mL), 80% NH_2_NH_2_·H_2_O (10 equiv) was slowly added at room temperature; then, the mixture was stirred for about 4~5 h, and the precipitate was gradually generated. When TLC indicated that the reaction was complete, the precipitate was filtered and dried in vacuo.

(S)-5-phenyl-3-tosyl-1,2,3,6-tetrahydroazepino [4,5-b]indole-2-carbohydrazide (**5a**):

White solid, yield 83%, mp 135–137 °C. ^1^H NMR (400 MHz, DMSO-d_6_) δ 10.04 (s, 1H), 9.12 (s, 1H), 7.73 (d, J = 7.2 Hz, 2H), 7.54−7.34 (m, 8H), 7.24 (d, J = 7.6 Hz, 1H), 7.05−6.82 (m, 3H), 5.27 (s, 1H), 3.98 (s, 2H), 3.79 (d, J = 11.2 Hz, 1H), 2.36 (s, 3H), 2.29 (d, J = 15.6 Hz, 1H). ^13^C NMR (100 MHz, DMSO-d_6_) δ 166.1, 144.3, 139.2, 135.8, 134.7, 131.1, 130.1, 129.1, 128.6, 127.9, 127.5, 127.0, 124.2, 121.2, 118.6, 117.4, 111.5, 110.3, 58.9, 26.9, 21.0. HRMS (ESI) calcd. for C_26_H_24_N_4_O_3_S (M+H)^+^ 473.1647, found 473.1639.

(S)-5-(4-methoxyphenyl)-3-tosyl-1,2,3,6-tetrahydroazepino [4,5-b]indole-2-carboh-ydrazide (**5b**):

Yellow solid, yield 35%, mp 150–152 °C. ^1^H NMR (400 MHz, DMSO-d_6_) δ 10.00 (s, 1H), 9.09 (s, 1H), 7.70 (d, J = 8.0 Hz, 2H), 7.39 (d, J = 7.6 Hz, 3H), 7.34 (d, J = 8.0 Hz, 2H), 7.22 (d, J = 7.6 Hz, 1H), 7.04 (d, J = 8.4 Hz, 2H), 6.95 (dt, J = 14.8, 6.8 Hz, 2H), 6.84 (s, 1H), 5.26 (s, 1H), 4.00 (s, 2H), 3.82 (s, 3H), 3.74 (dd, J = 15.6, 5.2 Hz, 1H), 2.35 (s, 3H), 2.28 (d, J = 14.8 Hz, 1H). ^13^C NMR (100 MHz, DMSO-d_6_) δ 166.2, 158.8, 144.2, 135.8, 134.8, 131.5, 131.3, 130.3, 130., 127.8, 126.9, 123.4, 121.2, 118.6, 117.6, 117.4, 114.0, 111.5, 110.1, 59.2, 55.2, 26.8, 21.0. HRMS (ESI) calcd. for C_27_H_26_N_4_O_4_S (M+H)^+^ 503.1753, found 503.1746.

(S)-5-(4-chlorophenyl)-3-tosyl-1,2,3,6-tetrahydroazepino [4,5-b]indole-2-carbohyd-razide (**5c**):

Yellow solid, yield 32%, mp 144–145 °C. ^1^H NMR (400 MHz, DMSO-d_6_) δ 10.07 (s, 1H), 9.09 (s, 1H), 7.74 (d, J = 8.4 Hz, 2H), 7.54 (d, J = 8.8 Hz, 2H), 7.43 (d, J = 8.4 Hz, 4H), 7.37 (d, J = 8.0 Hz, 1H), 7.19 (d, J = 7.6 Hz, 1H), 7.01−6.90 (m, 2H), 6.89 (s, 1H), 5.24 (d, J = 2.8 Hz, 1H), 3.95 (s, 2H), 3.78 (dd, J = 16.0, 5.2 Hz, 1H), 2.37 (s, 3H), 2.21 (dd, J = 16.0, 2.8 Hz, 1H). ^13^C NMR (100 MHz, DMSO-d_6_) δ 165.9, 144.4, 138.0, 135.8, 134.5, 132.2, 131.1, 130.8, 130.2, 128.6, 127.9, 127.1, 124.3, 121.3, 118.7, 117.4, 115.5, 111.4, 110.3, 58.4, 26.9, 21.0. HRMS (ESI) calcd. for C_26_H_23_ClN_4_O_3_S (M+H)^+^ 507.1257, found 507.1251.

#### 3.2.6. General Procedures for the Preparation of Compounds **6a**–**6c**

A mixture of **5** (1.0 equiv.) and p-chlorobenzaldehyde (1.5 equiv) in ethanol (30 mL) was heated to 100 °C. When TLC indicated that the reaction was complete, the mixture was concentrated in vacuo, and then purified by chromatography on a column of silica gel (petroleum ether: ethyl acetate = 2:1) to offer **6a**–**6c**.

(S)-N’-(4-chlorobenzylidene)-5-phenyl-3-tosyl-1,2,3,6-tetrahydroazepino [4,5-b]indole-2-carbohydrazide (**6a**):

Yellow solid 0.42 g, yield 83%, mp 147–149 °C. ^1^H NMR (400 MHz, DMSO-d_6_) δ 11.42 and 11.19 (s, 1H), 10.27 and 10.15 (s, 1H), 8.19 and 7.89 (s, 1H), 7.88−6.76 (m, 18H), 6.07−6.01 and 5.45−5.40 (m, 1H), 4.12 and 3.87 (dd, J = 15.6, 5.6 Hz, 1H), 2.75−2.67 and 2.46−2.42 (m, 1H), 2.40 and 2.37 (s, 3H). ^13^C NMR (100 MHz, DMSO-d_6_) δ 168.0, 163.9, 146.4, 145.0, 144.8, 142.8, 140.0, 139.6, 136.5, 136.4, 135.7, 135.2, 135.0, 134.9, 133.5, 133.4, 132.8, 131.9, 130.7, 129.7, 129.5, 129.3, 129.1, 129.0, 128.2, 128.1, 127.9, 127.8, 127.6, 127.5, 125.2, 124.6, 121.8, 121.5, 119.2, 119.1, 117.5, 116.7, 115.3, 112.1, 110.4, 109.9, 59.9, 27.6, 27.3, 21.6. HRMS (ESI) calcd. for C_33_H_27_ClN_4_O_3_S (M+H)^+^ 595.1570, found 595.1564.

(S)-N’-(4-chlorobenzylidene)-5-(4-methoxyphenyl)-3-tosyl-1,2,3,6-tetrahydroazepino [4,5-b]indole-2-carbohydrazide (**6b**):

Yellow solid 0.30 g, yield 60%, mp 240–242 °C. ^1^H NMR (400 MHz, DMSO-d_6_) δ 11.44 and 11.21 (s, 1H), 10.26 and 10.14 (s, 1H), 8.22 and 7.91 (s, 1H), 7.89−6.77 (m, 17H), 6.07 and 5.44 (s, 1H), 4.12 (dd, J = 15.2, 5.6 Hz, 1H), 3.83 (s, 3H), 2.72 and 2.45 (d, J = 14.8 Hz, 1H), 2.40 and 2.37 (s, 3H). ^13^C NMR (100 MHz, DMSO-d_6_) δ 168.1, 164.0, 163.2, 159.4, 159.2, 144.7, 142.7, 136.42, 136.37, 135.8, 135.0, 134.9, 133.5, 133.4, 133.2, 132.3, 132.2, 131.7, 130.7, 130.6, 130.5, 129.7, 129.3, 129.1, 129.0, 128.1, 127.8, 127.5, 127.4, 124.3, 123.8, 121.7, 121.4, 119.2, 119.1, 117.9, 117.58, 117.56, 116.7, 115.5, 114.7, 112.1, 110.2, 109.7, 60.1, 59.7, 56.5, 55.7, 27.5, 27.3, 21.6, 19.1. HRMS (ESI) calcd. for C_34_H_29_ClN_4_O_4_S (M+H)^+^ 625.1676, found 625.1674.

(S)-N’-(4-chlorobenzylidene)-5-(4-chlorophenyl)-3-tosyl-1,2,3,6-tetrahydroazepino [4,5-b]indole-2-carbohydrazide (**6c**):

Yellow solid 0.39 g, yield 79%, mp 153–155 °C. ^1^H NMR (400 MHz, DMSO-d_6_) δ 11.42 and 11.19 (s, 1H), 10.33 and 10.21 (s, 1H), 8.25−6.76 (m, 18H), 6.05 (d, J = 2.8 Hz, 0.6H) and 5.44 (s, 0.4H), 4.15 and 3.91 (dd, J = 15.6, 5.6 Hz, 1H), 2.70 (d, J = 14.0 Hz, 1H), 2.41 and 2.38 (s, 3H). ^13^C NMR (100 MHz, DMSO-d_6_) δ 167.8, 163.7, 146.5, 145.1, 144.9, 142.8, 138.9, 138.5, 136.5, 136.4, 135.6, 135.04, 134.98, 133.5, 133.4, 132.8, 132.5, 131.7, 131.5, 131.1, 130.7, 129.7, 129.3, 129.2, 129.1, 129.0, 128.2, 127.8, 127.6, 127.5, 125.3, 124.7, 121.9, 121.6, 119.3, 119.2, 117.6, 116.7, 115.8, 113.8, 112.0, 110.4, 109.9, 59.5, 59.4, 27.7, 27.4, 21.6. HRMS (ESI) calcd. for C_33_H_26_ClN_4_O_3_S (M+H)^+^ 629.1181, found 629.1171.

#### 3.2.7. General Procedures for the Preparation of Compounds **6d**–**6aa**

To a solution of **5a** (0.4 g, 0.85 mmol) in ethanol (30 mL), the corresponding aldehyde (1.5 equiv) was added, and then the mixture was heated to 100 °C. When TLC indicated that the reaction was complete, the mixture was concentrated in vacuo, and then purified by chromatography on a column of silica gel (petroleum ether: ethyl acetate = 2:1) to offer **6d**–**6aa**.

(S)-N’-benzylidene-5-phenyl-3-tosyl-1,2,3,6-tetrahydroazepino [4,5-b]indole-2-carbohydrazide (**6d**):

Yellow solid 0.41 g, yield 88%, mp 222–224 °C. ^1^H NMR (400 MHz, DMSO-d_6_) δ 11.35 and 11.12 (s, 1H, Ar-NH), 10.28 and 10.15 (s, 1H), 8.21−6.72 (m, 20H), 6.04 and 5.42 (m, 1H), 4.14 and 3.88 (dd, J = 15.6, 5.6 Hz, 1H), 2.70 and 2.39 (dd, J = 15.6, 5.6 Hz, 1H), 2.40 and 2.38 (s, 3H). ^13^C NMR (100 MHz, DMSO-d_6_) δ 167.9, 163.7, 147.7, 145.0, 144.8, 144.0, 140.0, 139.6, 136.5, 136.4, 135.7, 135.2, 134.6, 134.4, 133.4, 132.8, 131.9, 130.7, 130.6, 130.5, 129.7, 129.6, 129.5, 129.3, 129.2, 129.1, 128.2, 128.1, 127.9, 127.8, 127.6, 127.49, 127.45, 127.4, 125.2, 124.6, 121.7, 121.5, 119.2, 119.1, 117.5, 117.4, 116.6, 115.2, 112.1, 110.3, 109.9, 59.9, 59.8, 27.6, 27.2, 22.7, 21.6. HRMS (ESI) calcd. for C_33_H_28_N_4_O_3_S (M+H)^+^ 561.1960, found 561.1954.

(S)-N’-(4-nitrobenzylidene)-5-phenyl-3-tosyl-1,2,3,6-tetrahydroazepino [4,5-b]indole-2-carbohydrazide (**6e**):

Yellow solid 0.43 g, yield 84%, mp 155–157 °C. ^1^H NMR (400 MHz, DMSO-d_6_) δ 11.69 and 11.44 (s, 1H), 10.29 and 10.16 (s, 1H), 8.42−6.74 (m, 19H), 6.08 and 5.45 (dd, J = 4.8, 2.4 Hz, 1H), 4.13 and 3.89 (dd, J = 16.0, 5.6 Hz, 1H), 2.72 (dd, J = 16.0, 2.4 Hz, 1H), 2.40 and 2.38 (s, 3H). ^13^C NMR (100 MHz, DMSO-d_6_) δ 167.7, 163.7, 147.9, 147.8, 144.7, 144.5, 144.4, 141.3, 140.2, 139.5, 139.1, 136.0, 135.9, 135.1, 134.6, 132.2, 131.4, 130.2, 129.0, 128.8, 127.9, 127.8, 127.6, 127.4, 127.2, 127.0, 126.9, 124.6, 124.3, 124.0, 123.9, 121.3, 121.0, 118.71, 118.67, 117.04, 117.01, 116.2, 114.8, 111.6, 109.8, 109.3, 59.4, 59.3, 27.1, 26.9, 21.1. HRMS (ESI) calcd. for C_33_H_27_N_5_O_5_S (M+H)^+^ 606.1811, found 606.1809.

(S)-N’-(4-methoxybenzylidene)-5-phenyl-3-tosyl-1,2,3,6-tetrahydroazepino [4,5-b]indole-2-carbohydrazide (**6f**):

Yellow solid 0.46 g, yield 92%, mp 232–234 °C. ^1^H NMR (400 MHz, DMSO-d_6_) δ 11.20 and 10.99 (s, 1H), 10.28 and 10.15 (s, 1H), 8.11 and 7.82 (s, 1H), 7.77 (t, J = 8.8 Hz, 3H), 7.52−6.75 (m, 15H), 6.01 and 5.39 (s, 1H), 4.14 (dd, J = 15.6, 5.6 Hz, 1H), 3.86 and 3.76 (s, 3H), 2.67 (d, J = 14.8 Hz, 1H), 2.40 and 2.38 (s, 3H). ^13^C NMR (100 MHz, DMSO-d_6_) δ 167.2, 163.04, 163.01, 160.9, 160.8, 147.2, 144.5, 144.4, 143.4, 139.6, 139.2, 136.0, 135.3, 134.7, 132.3, 131.5, 130.3, 129.6, 129.1, 128.8, 128.6, 128.5, 127.7, 127.6, 127.6, 127.4, 127.4, 127.1, 127.0, 126.7, 126.5, 126.5, 124.8, 124.2, 121.3, 121.0, 118.74, 118.66, 117.11, 117.08, 116.9, 116.2, 114.7, 114.6, 114.2, 111.7, 110.0, 109.5, 59.5, 59.3, 55.4, 55.3, 27.2, 26.8, 21.1. HRMS (ESI) calcd. for C_34_H_30_N_4_O_4_S (M+H)^+^ 591.2066, found 591.2066.

(S)-N’-(3-methoxybenzylidene)-5-phenyl-3-tosyl-1,2,3,6-tetrahydroazepino [4,5-b]indole-2-carbohydrazide (**6g**):

Yellow solid 0.40 g, yield 80%, mp 230–232 °C. ^1^H NMR (400 MHz, DMSO-d_6_) δ 11.38 and 11.19 (s, 1H), 10.30 and 10.17 (s, 1H), 8.18 and 7.89 (s, 1H), 7.84−6.75 (m, 18H), 6.04 and 5.45 (s, 1H), 4.10 and 3.92 (dd, J = 15.2, 5.2 Hz, 1H), 3.86 and 3.73 (s, 3H), 2.70 and 2.45 (d, J = 15.2 Hz, 1H), 2.38 (d, J = 6.0 Hz, 3H). ^13^C NMR (100 MHz, DMSO-d_6_) δ 168.0, 163.7, 160.2, 159.9, 147.6, 145.0, 144.8, 143.7, 140.0, 139.7, 136.5, 136.4, 136.0, 135.9, 135.6, 135.2, 132.8, 132.0, 130.7, 130.7, 130.3, 129.6, 129.2, 128.2, 128.1, 127.9, 127.8, 127.6, 127.5, 125.2, 124.6, 121.8, 121.5, 120.6, 119.9, 119.1, 117.5, 117.3, 116.9, 116.7, 116.4, 115.7, 112.2, 112.1, 111.2, 110.3, 110.0, 60.1, 59.7, 55.6, 55.5, 27.7, 27.1, 21.6. HRMS (ESI) calcd. for C_34_H_30_N_4_O_4_S (M+H)^+^ 591.2066, found 591.2063.

(S)-N’-(2-methoxybenzylidene)-5-phenyl-3-tosyl-1,2,3,6-tetrahydroazepino [4,5-b]indole-2-carbohydrazide (**6h**):

Yellow solid 0.42 g, yield 84%, mp 134–136 °C. ^1^H NMR (400 MHz, DMSO-d_6_) δ 11.38 and 11.10 (s, 1H), 10.29 and 10.16 (s, 1H), 8.57 and 8.24 (s, 1H), 8.06−6.75 (m, 18H), 6.05 and 5.40 (d, J = 3.0 Hz, 1H), 4.16 (dd, J = 15.6, 5.6 Hz, 0.6 H) and 3.91 (dd, J = 9.6, 5.6Hz, 0.4H), 3.86 and 3.85 (s, 3H), 2.71 and 2.42 (dd, J = 15.2, 2.0Hz, 1H), 2.39 and 2.37 (s, 3H). ^13^C NMR (100 MHz, DMSO-d_6_) δ 167.8, 163.6, 158.2, 158.1, 144.8, 143.2, 140.0, 139.8, 139.6, 136.5, 136.4, 135.7, 135.2, 132.8, 132.0, 131.9, 130.8, 130.7, 129.6, 129.3, 128.2, 128.1, 127.9, 127.8, 127.6, 127.5, 125.9, 125.8, 125.2, 124.7, 122.6, 122.4, 121.8, 121.54, 121.49, 121.1, 119.2, 119.1, 117.6, 117.5, 116.7, 115.3, 112.5, 112.2, 112.1, 110.5, 109.9, 59.9, 59.8, 56.2, 56.1, 27.5, 27.2, 21.6. HRMS (ESI) calcd. for C_34_H_30_N_4_O_4_S (M+H)^+^ 591.2066, found 591.2063.

(S)-N’-(3-nitrobenzylidene)-5-phenyl-3-tosyl-1,2,3,6-tetrahydroazepino [4,5-b]indole-2-carbohydrazide (**6i**):

Deep-yellow solid 0.43 g, yield 84%, mp 249–252 °C. ^1^H NMR (400 MHz, DMSO-d_6_) δ 11.66 and 11.43 (s, 1H), 10.31 and 10.18 (s, 1H), 8.68−6.73 (m, 19H), 6.11 and 5.49 (s, 1H), 4.08 and 3.92 (d, J = 12.0 Hz, 1H), 2.70 and 2.45 (d, J = 15.2 Hz, 1H), 2.40 and 2.38 (s, 3H). ^13^C NMR (100 MHz, DMSO-d_6_) δ 168.3, 164.2, 148.9, 148.6, 145.3, 145.0, 144.9, 141.8, 139.9, 139.6, 136.5, 136.4, 136.3, 136.2, 135.5, 135.2, 133.7, 133.4, 132.8, 132.0, 131.1, 130.8, 130.7, 129.5, 129.3, 128.1, 127.9, 127.8, 127.5, 127.4, 125.1, 124.8, 124.7, 124.6, 121.8, 121.7, 121.5, 121.4, 119.2, 117.5, 116.7, 115.7, 112.1, 110.3, 109.9, 59.9, 27.7, 27.2, 21.6. HRMS (ESI) calcd. for C_33_H_27_N_5_O_5_S (M+H)^+^ 606.1811, found 606.1806.

(S)-N’-(4-cyanobenzylidene)-5-phenyl-3-tosyl-1,2,3,6-tetrahydroazepino [4,5-b]indole-2-carbohydrazide (**6j**):

Yellow solid 0.41 g, yield 83%, mp 157–159 °C. ^1^H NMR (400 MHz, DMSO-d_6_) δ 11.64 and 11.40 (s, 1H), 10.31 and 10.18 (s, 1H), 8.31−6.75 (m, 19H), 6.10 and 5.48 (s, 1H), 4.14 and 3.92 (dd, J = 15.2, 5.2 Hz, 1H), 2.75 and 2.47 (d, J = 15.2 Hz, 1H), 2.40 and 2.38 (s, 3H). ^13^C NMR (100 MHz, DMSO-d_6_) δ 168.2, 164.1, 145.8, 145.0, 144.9, 142.2, 140.0, 139.6, 139.0, 138.9, 136.5, 135.7, 135.1, 133.5, 133.1, 132.8, 131.9, 130.7, 129.5, 129.3, 128.1, 128.0, 127.9, 127.7, 127.5, 125.1, 124.5, 121.8, 121.5, 119.22, 119.17, 117.5, 116.7, 115.3, 112.42, 112.36, 112.1, 110.3, 109.8, 59.9, 27.6, 27.4, 21.6. HRMS (ESI) calcd. for C_34_H_27_N_5_O_3_S (M+H)^+^ 586.1913, found 586.1914.

(S)-N’-(4-tert-butylbenzylidene)-5-phenyl-3-tosyl-1,2,3,6-tetrahydroazepino [4,5-b]indole-2-carbohydrazide (**6k**):

Yellow solid 0.43 g, yield 83%, mp 150–152 °C. ^1^H NMR (400 MHz, DMSO-d_6_) δ 11.28 and 11.07 (s, 1H), 10.28 and 10.15 (s, 1H), 8.15 and 7.86 (s, 1H), 7.81−6.77 (m, 18H), 6.02 and 5.40 (d, J = 2.8 Hz, 1H), 4.12 and 3.87 (dd, J = 15.6, 5.6 Hz, 1H), 2.67 (d, J = 13.6 Hz, 1H), 2.40 and 2.38 (s, 3H), 1.35 and 1.25 (s, 9H). ^13^C NMR (100 MHz, DMSO-d_6_) δ 167.9, 163.6, 153.3, 147.7, 144.9, 144.8, 143.9, 140.0, 139.6, 136.5, 135.7, 135.2, 132.8, 131.9, 131.9, 131.7, 130.7, 129.5, 129.3, 128.2, 128.1, 127.8, 127.6, 127.5, 127.3, 127.2, 126.4, 126.0, 125.2, 124.6, 121.8, 121.5, 119.2, 119.1, 117.5, 116.6, 115.3, 112.1, 110.4, 109.9, 59.9, 35.1, 35.0, 31.5, 27.7, 27.2, 21.6. HRMS (ESI) calcd. for C_37_H_36_N_4_O_3_S (M+H)^+^ 617.2586, found 617.2578.

(S)-N’-(4-(dimethylamino)benzylidene)-5-phenyl-3-tosyl-1,2,3,6-tetrahydroazepino [4,5-b]indole-2-carbohydrazide (**6l**):

Yellow solid 0.35 g, yield 68%, mp 146–148 °C. ^1^H NMR (400 MHz, DMSO-d_6_) δ 11.01 and 10.83 (s, 1H), 10.27 and 10.15 (s, 1H), 8.06−6.61 (m, 19H), 6.02 and 5.40 (s, 1H), 4.19 and 3.88 (d, J = 11.2 Hz, 1H), 3.01 and 2.91 (s, 6H), 2.68 (d, J = 15.2 Hz, 1H), 2.39 and 2.37 (s, 3H). ^13^C NMR (100 MHz, DMSO-d_6_) δ 167.4, 163.2, 152.0, 144.9, 144.8, 144.7, 140.1, 139.7, 136.5, 136.4, 135.8, 135.2, 132.8, 132.0, 130.7, 129.5, 129.3, 128.8, 128.6, 128.2, 128.1, 127.9, 127.6, 127.5, 125.3, 124.7, 122.0, 121.73, 121.69, 121.5, 119.2, 119.1, 117.6, 117.5, 116.7, 115.3, 112.5, 112.1, 110.5, 110.1, 60.0, 59.8, 40.3, 27.8, 27.1, 21.6. HRMS (ESI) calcd. for C_35_H_33_N_5_O_3_S (M+H)^+^ 604.2382, found 604.2382.

(S)-N’-(biphenyl-4-ylmethylene)-5-phenyl-3-tosyl-1,2,3,-tetrahydroazepino [4,5-b]indole-2-carbohydrazide (**6m**):

Yellow solid 0.42 g, yield 78%, mp 153–155 °C. ^1^H NMR (400 MHz, DMSO-d_6_) δ 11.42 and 11.20 (s, 1H), 10.31 and 10.19 (s, 1H), 8.25 and 7.96 (s, 1H), 7.94−6.78 (m, 23H), 6.15−6.04, 5.50−5.37 (m, 1H), 4.19 and 3.92 (dd, J = 15.6, 5.6 Hz, 1H), 2.74, 2.46 (d, J =14.0 Hz, 1H), 2.40 and 2.38 (s, 3H). ^13^C NMR (100 MHz, DMSO-d_6_) δ 167.4, 163.3, 146.8, 144.5, 144.4, 143.1, 141.6, 141.5, 139.5, 139.4, 139.2, 139.1, 136.0, 135.9, 135.2, 134.7, 133.2, 133.0, 132.3, 131.5, 130.2, 129.1, 129.0, 128.8, 128.7, 127.92, 127.85, 127.7, 127.6, 127.5, 127.3, 127.3, 127.1, 127.0, 126.9, 126.7, 126.6, 124.7, 121.0, 118.71, 118.66, 117.1, 117.0, 116.2, 114.7, 111.6, 109.9, 109.4, 59.4, 27.1, 26.8, 21.1. HRMS (ESI) calcd. for C_39_H_32_N_4_O_3_S (M+H)^+^ 637.2273, found 637.2267.

(S)-N’-(3-chlorobenzylidene)-5-phenyl-3-tosyl-1,2,3,6-tetrahydroazepino [4,5-b]indole-2-carbohydrazide (**6n**):

Yellow solid 0.39 g, yield 79%, mp 247–249 °C. ^1^H NMR (400 MHz, DMSO-d_6_) δ 11.53 and 11.30 (s, 1H), 10.31 and 10.18 (s, 1H), 8.20 and 7.91 (s, 1H), 7.89−6.78 (m, 18H), 6.08 and 5.46 (s, 1H), 4.07 and 3.91 (dd, J = 15.2, 5.2 Hz, 1H), 2.74 (d, J = 15.2 Hz, 0.6H) and 2.45 (d, J = 17.6 Hz, 0.4H), 2.39 and 2.37 (s, 3H). ^13^C NMR (100 MHz, DMSO-d_6_) δ 167.7, 163.5, 145.5, 144.5, 144.3, 142.0, 139.4, 139.1, 136.3, 136.2, 136.0, 135.9, 135.1, 134.7, 133.9, 133.6, 132.3, 131.5, 131.0, 130.6, 130.2, 130.2, 129.7, 129.6, 129.0, 128.8, 128.7, 127.7, 127.6, 127.4, 127.3, 127.0, 126.3, 126.2, 125.7, 125.5, 124.7, 124.1, 121.3, 121.0, 118.71, 118.67, 117.04, 116.98, 116.2, 115.2, 111.6, 109.8, 109.5, 59.6, 59.3, 27.1, 26.7, 21.1. HRMS (ESI) calcd. for C_33_H_27_ClN_4_O_3_S (M+H)^+^ 595.1570, found 595.1563.

(S)-N’-(2-chlorobenzylidene)-5-phenyl-3-tosyl-1,2,3,6-tetrahydroazepino [4,5-b]indole-2-carbohydrazide (**6o**):

Yellow solid 0.46 g, yield 92%, mp 132–134 °C. ^1^H NMR (400 MHz, DMSO-d_6_) δ 11.63 and 11.33 (s, 1H), 10.31 and 10.17 (s, 1H), 8.62 and 8.31 (s, 1H), 8.25−6.76 (m, 18H), 6.08 and 5.46 (s, 1H), 4.14 (d, J = 11.8 Hz, 0.6H) and 3.93 (d, J = 13.2 Hz, 0.4H), 2.74 and 2.44 (d, J = 15.2 Hz, 1H), 2.39 (s, 3H). ^13^C NMR (100 MHz, DMSO-d_6_) δ 168.0, 163.9, 145.0, 144.8, 143.8, 140.3, 140.0, 139.6, 136.5, 136.5, 135.7, 135.1, 133.6, 132.8, 132.0, 131.9, 131.8, 131.7, 130.7, 130.6, 130.3, 129.5, 129.3, 128.5, 128.2, 128.1, 127.94, 127.87, 127.8, 127.6, 127.5, 127.4, 127.2, 125.2, 124.5, 121.8, 121.6, 119.2, 117.6, 116.7, 115.4, 112.1, 110.5, 109.9, 59.9, 27.5, 27.4, 21.6. HRMS (ESI) calcd. for C_33_H_27_ClN_4_O_3_S (M+H)^+^ 595.1570, found 595.1568.

(S)-N’-(2,4-dichlorobenzylidene)-5-phenyl-3-tosyl-1,2,3,6-tetrahydroazepino [4,5-b]indole-2-carbohydrazide (**6p**):

Yellow solid 0.39 g, yield 74%, mp 140–142 °C. ^1^H NMR (400 MHz, DMSO-d_6_) δ 11.65 and 11.36 (s, 1H), 10.29 and 10.16 (s, 1H), 8.57 and 8.24 (s, 1H), 8.22−6.77 (m, 17H), 6.07 and 5.45 (s, 1H), 4.16−4.05 and 3.96−3.85 (m, 1H), 2.73 and 2.42 (d, J = 15.2 Hz, 1H), 2.39 and 2.37 (s, 3H). ^13^C NMR (100 MHz, DMSO-d_6_) δ 168.0, 164.0, 145.0, 144.9, 142.8, 140.0, 139.6, 139.4, 136.5, 136.4, 135.7, 135.6, 135.6, 135.1, 134.2, 132.8, 131.8, 130.9, 130.8, 130.7, 130.1, 129.8, 129.5, 129.3, 128.8, 128.6, 128.4, 128.3, 128.2, 128.1, 127.9, 127.7, 127.60, 127.5, 125.1, 124.5, 121.8, 121.6, 119.2, 117.70, 117.65, 116.7, 115.4, 112.1, 110.5, 109.8, 60.0, 59.8, 27.4, 21.6. HRMS (ESI) calcd. for C_33_H_26_Cl_2_N_4_O_3_S (M+H)^+^ 629.1181, found 629.1175.

(S)-N’-(3,4-dichlorobenzylidene)-5-phenyl-3-tosyl-1,2,3,6-tetrahydroazepino [4,5-b]indole-2-carbohydrazide (**6q**):

Yellow solid 0.48 g, yield 75%, mp 144–146 °C. ^1^H NMR (400 MHz, DMSO-d_6_) δ 11.59 and 11.33 (s, 1H), 10.30 and 10.17 (s, 1H), 8.18 and 8.04 (s, 1H), 7.91−6.77 (m, 17H), 6.06 and 5.45 (d, J = 2.8 Hz, 1H), 4.06 and 3.89 (dd, J = 15.6, 5.6 Hz, 1H), 2.72 and 2.44 (d, J = 14.0 Hz, 1H), 2.39 and 2.37 (s, 3H). ^13^C NMR (100 MHz, DMSO-d_6_) δ 167.7, 163.5, 144.5, 144.4, 141.0, 139.4, 139.1, 136.0, 135.9, 135.1, 134.9, 134.8, 134.7, 132.3, 132.2, 131.9, 131.6, 131.3, 130.9, 130.2, 130.2, 129.0, 128.8, 128.7, 128.6, 128.4, 127.6, 127.5, 127.4, 127.2, 127.0, 126.8, 126.5, 124.6, 124.1, 121.3, 121.0, 118.7, 117.0, 116.9, 116.2, 115.2, 111.6, 109.8, 109.4, 59.5, 59.3, 27.1, 26.8, 21.1. HRMS (ESI) calcd. for C_33_H_26_Cl_2_N_4_O_3_S (M+H)^+^ 629.1181, found 629.1178.

(S)-N’-(3,4-dimethoxybenzylidene)-5-phenyl-3-tosyl-1,2,3,6-tetrahydroazepino [4,5-b]indole-2-carbohydrazide (**6r**):

Yellow solid 0.46 g, yield 87%, mp 146–148 °C. ^1^H NMR (400 MHz, DMSO-d_6_) δ 11.19 and 11.04 (s, 1H), 10.27 and 10.13 (s, 1H), 8.09 and 7.81 (s, 1H), 7.80−6.76 (m, 17H), 6.01−5.95 and 5.41−5.37 (m, 1H), 4.06 (dd, J = 15.2, 5. 6Hz, 1H), 3.87 and 3.76 (s, 3H), 3.85 and 3.71 (s, 3H), 2.61 and 2.41 (dd, J = 15.6, 2.4 Hz, 1H), 2.39 (s, 3H). ^13^C NMR (100 MHz, DMSO-d_6_) δ 167.9, 163.4, 151.2, 151.1, 149.6, 149.4, 147.9, 145.0, 144.8, 143.8, 139.9, 139.7, 136.5, 136.4, 135.6, 135.2, 132.7, 132.0, 130.7, 130.6, 129.6, 129.3, 129.2, 128.2, 128.1, 127.9, 127.8, 127.6, 127.5, 127.4, 127.1, 125.2, 124.6, 122.5, 121.7, 121.5, 121.3, 119.2, 119.1, 117.5, 117.2, 116.7, 116.1, 112.3, 112.14, 112.10, 111.7, 110.3, 110.2, 109.2, 108.2, 60.2, 59.5, 56.1, 56.0, 55.8, 55.7, 27.7, 27.0, 21.5. HRMS (ESI) calcd. for C_35_H_32_N_4_O_5_S (M+H)^+^ 621.2171, found 621.2165.

(S)-N’-(benzo[d][1,3]dioxol-5-ylmethylene)-5-phenyl-3-tosyl-1,2,3,6-tetrahydroazepino [4,5-b]indole-2-carbohydrazide (**6s**):

Yellow solid 0.44 g, yield 87%, mp 143–145 °C. ^1^H NMR (400 MHz, DMSO-d_6_) δ 11.23 and 11.00 (s, 1H), 10.26 and 10.14 (s, 1H), 8.10−6.76 (m, 18H), 6.16 and 6.03 (d, J = 2.8 Hz, 1H), 6.03−5.99 and 5.41−5.37 (m, 1H), 4.10 and 3.86 (dd, J = 15.6, 5.6 Hz, 1H), 2.68 and 2.41 (dd, J = 15.6, 2.4 Hz, 1H), 2.39 and 2.37 (s, 3H). ^13^C NMR (100 MHz, DMSO-d_6_) δ 167.8, 163.6, 149.6, 149.5, 148.7, 148.4, 147.6, 144.9, 144.8, 143.7, 140.0, 139.6, 136.5, 136.4, 135.7, 135.2, 132.7, 131.9, 130.7, 130.6, 129.5, 129.3, 129.2, 129.0, 128.8, 128.2, 128.1, 127.9, 127.8, 127.6, 127.5, 125.2, 124.6, 123.8, 123.6, 121.8, 121.5, 119.2, 119.1, 117.6, 117.5, 116.7, 115.3, 112.1, 110.4, 110.0, 109.1, 108.8, 105.5, 105.5, 102.1, 102.0, 60.0, 59.8, 27.7, 27.2, 21.6. HRMS (ESI) calcd. for C_34_H_28_N_4_O_5_S (M+H)^+^ 605.1858, found 605.1852.

(S)-N’-((2,3-dihydrobenzo[b][1,4]dioxin-6-yl)methylene)-5-phenyl-3-tosyl-1,2,3,6-tetrahydroazepino [4,5-b]indole-2-carbohydrazide (**6t**):

Yellow solid 0.35 g, yield 85%, mp 148–150 °C. ^1^H NMR (400 MHz, DMSO-d_6_) δ 11.23 and 11.02 (s, 1H), 10.28 and 10.16 (s, 1H), 8.07−6.77 (m, 18H), 6.01 and 5.40 (s, 1H), 4.34 (s, 3H), 4.18 (s, 1H), 4.09 and 3.87 (dd, J = 15.6, 5.6 Hz, 1H), 2.69 and 2.41 (d, J = 14.0 Hz, 1H), 2.40 and 2.37 (s, 3H). ^13^C NMR (100 MHz, DMSO-d_6_) δ 172.5, 168.3, 152.2, 150.5, 150.4, 149.7, 149.6, 149.0, 148.7, 148.3, 144.8, 144.4, 141.2, 141.1, 140.4, 139.9, 137.5, 136.7, 135.5, 134.3, 134.0, 132.9, 132.82, 132.78, 132.6, 132.5, 132.3, 132.2, 130.0, 129.3, 126.5, 126.2, 125.9, 125.8, 123.9, 123.8, 123.0, 122.6, 122.3, 122.2, 121.4, 120.4, 120.2, 120.0, 116.8, 115.1, 114.7, 69.6, 69.5, 69.3, 69.2, 64.7, 64.6, 32.4, 31.9, 26.3. HRMS (ESI) calcd. for C_35_H_30_N_4_O_5_S (M+H)^+^ 619.2015, found 619.2018.

(S)-N’-(3,5-di-tert-butyl-4-hydroxybenzylidene)-5-phenyl-3-tosyl-1,2,3,6-tetrahydroazepino [4,5-b]indole-2-carbohydrazide (**6u**):

Yellow solid 0.50 g, yield 86%, mp 160–162 °C. ^1^H NMR (400 MHz, DMSO-d_6_) δ 11.09 and 11.04 (s, 1H), 10.32 and 10.16 (s, 1H), 8.10 and 7.82 (s, 1H), 7.78 and 7.74 (d, J = 8.4 Hz, 2H), 7.59−6.79 (m, 15H), 5.94 (s, 1H), 5.42 (s, 1H), 4.08−3.99 and 3.93−3.85 (m, 1H), 2.53 and 2.42 (s, 1H), 2.40 and 2.38 (s, 3H), 1.47 and 1.36 (s, 18H). ^13^C NMR (100 MHz, DMSO-d_6_) δ 167.9, 163.3, 156.6, 156.5, 149.3, 144.9, 144.8, 144.5, 139.9, 139.8, 139.7, 139.6, 136.5, 136.4, 135.2, 135.1, 132.8, 131.9, 130.7, 130.7, 129.6, 129.3, 128.2, 128.0, 127.9, 127.7, 127.6, 127.4, 126.1, 125.7, 125.2, 124.6, 124.3, 124.0, 121.7, 121.5, 119.2, 117.5, 117.1, 116.6, 116.0, 112.2, 112.1, 110.3, 110.0, 60.2, 59.4, 35.1, 34.9, 30.6, 26.5, 21.6, 21.5. HRMS (ESI) calcd. for C_41_H_44_N_4_O_4_S (M+H)^+^ 689.3161, found 689.3163.

(S)-N’-(4-bromo-2,6-difluorobenzylidene)-5-phenyl-3-tosyl-1,2,3,6-tetrahydroazepino [4,5-b]indole-2-carbohydrazide (**6v**):

Yellow solid 0.27 g, yield 47%, mp 199–201 °C. ^1^H NMR (400 MHz, DMSO-d_6_) δ 11.55 and 11.34 (s, 1H), 10.30 and 10.16 (s, 1H), 8.33 and 7.96 (s, 1H), 7.80−6.80 (m, 16H), 6.01−5.86 and 5.47−5.40 (m, 1H), 4.11 (dd, J = 15.6., 5.6 Hz, 1H), 2.57 (dd, J = 15.2, 1.6 Hz, 1H), 2.40 and 2.38 (s, 3H). ^13^C NMR (101 MHz, DMSO) δ 167.6, 163.3, 160.2 (d, J = 256 Hz), 160.1 (d, J = 256 Hz), 144.5, 144.4, 137.32, 136.0, 134.9, 134.6, 132.8, 132.3, 130.2, 129.0, 128.8, 127.63, 127.60, 127.4, 127.3, 127.1, 126.9, 124.6, 124.0, 122.7 (t, J = 13.0 Hz), 121.3, 121.0, 118.8, 116.5, 116.3, 116.1, 114.6, 111.6, 111.0 (t, J = 13.6 Hz), 109.9, 109.2, 59.41, 59.2, 26.9, 26.2, 21.1. HRMS (ESI) calcd. for C_33_H_25_BrF_2_N_4_O_3_S (M+H)^+^ 675.0877, found 675.0871.

(S)-5-phenyl-N’-(pyridin-3-ylmethylene)-3-tosyl-1,2,3,6-tetrahydroazepino [4,5-b]indole-2-carbohydrazide (**6w**):

Yellow solid 0.35 g, yield 73%, mp 152–154 °C. ^1^H NMR (400 MHz, DMSO-d_6_) δ 11.56 and 11.31 (s, 1H), 10.29 and 10.17 (s, 1H), 9.05−6.73 (m, 19H), 6.09, 5.46 (s, 1H), 4.15 and 3.91 (dd, J = 15.6, 4.0 Hz, 1H), 2.73 (d, J = 15.2 Hz, 0.6H) and 2.45 (d, J = 16.4 Hz, 0.4H), 2.39 and 2.37 (s, 3H). ^13^C NMR (100 MHz, DMSO-d_6_) δ 168.1, 164.0, 151.2, 149.1, 149.0, 145.1, 145.0, 144.8, 141.3, 140.0, 139.6, 136.5, 136.4, 135.7, 135.2, 134.0, 133.8, 132.7, 132.0, 130.7, 130.5, 130.4, 129.5, 129.3, 128.2, 128.1, 127.9, 127.8, 127.6, 127.5, 125.2, 124.7, 124.6, 124.3, 121.8, 121.5, 119.2, 119.1, 117.6, 117.5, 116.7, 115.5, 112.1, 110.3, 110.0, 60.0, 59.9, 27.6, 27.3, 21.6. HRMS (ESI) calcd. for C_32_H_27_N_5_O_3_S (M+H)^+^ 562.1913, found 562.1911.

(S)-5-phenyl-N’-(thiophen-2-ylmethylene)-3-tosyl-1,2,3,6-tetrahydroazepino [4,5-b]indole-2-carbohydrazide (**6x**):

Brown solid, yield 79%, mp 222–224 °C. ^1^H NMR (400 MHz, DMSO-d_6_) δ 11.32 and 11.15 (s, 1H), 10.32 and 10.17 (s, 1H), 8.42 and 8.08 (s, 1H), 7.83−6.74 (m, 17H), 6.00−5.79 and 5.46−5.35 (m, 1H), 4.11 (dd, J = 15.4, 5.5 Hz, 0.6H) and 3.89 (dd, J = 15.7, 5.4 Hz, 0.4H), 2.64 (d, J = 14.8 Hz, 1H), 2.40 and 2.37 (s, 3H). ^13^C NMR (100 MHz, DMSO-d_6_) δ 167.6, 163.6, 145.0, 144.9, 143.0, 140.0, 139.6, 139.5, 139.2, 138.8, 136.5, 136.4, 135.5, 135.1, 132.8, 131.9, 131.4, 131.1, 130.7, 129.6, 129.5, 129.3, 129.1, 128.7, 128.2, 128.1, 128.0, 127.9, 127.7, 127.6, 127.4, 125.2, 124.6, 121.8, 121.5, 119.2, 117.6, 116.6, 115.3, 112.1, 110.3, 109.9, 59.9, 59.7, 27.6, 27.1, 21.6. HRMS (ESI) calcd. for C_31_H_26_N_4_O_3_S_2_ (M+H)^+^ 567.1524, found 567.1526.

(S)-N’-((1H-pyrrol-2-yl)methylene)-5-phenyl-3-tosyl-1,2,3,6-tetrahydroazepino [4,5-b]indole-2-carbohydrazide (**6y**):

Yellow solid 0.33 g, yield 72%, mp 154–156 °C. ^1^H NMR (400 MHz, DMSO-d_6_) δ 11.64 and 11.31 (s, 1H), 10.98 and 10.72 (s, 1H), 10.24 and 10.14 (s, 1H), 8.02 and 7.71(s, 1H), 7.81 and 7.77 (d, J = 8.0 Hz, 2H), 7.58−6.74 (m, 13H), 6.51 and 6.38 (s, 1H), 6.22 and 5.38 (s, 1H), 6.07 (s, 1H), 4.34 and 3.87 (dd, J = 15.6, 5.6 Hz, 1H), 2.66 (d, J = 15.2 Hz, 1H), 2.39 and 2.37 (s, 3H). ^13^C NMR (100 MHz, DMSO-d_6_) δ 167.4, 163.0, 144.9, 144.7, 141.1, 140.2, 139.7, 136.7, 136.5, 136.4, 136.1, 135.2, 132.8, 131.9, 130.7, 130.6, 129.5, 129.2, 128.3, 128.1, 128.0, 127.8, 127.6, 127.5, 127.2, 125.5, 124.7, 122.9, 122.7, 121.7, 121.4, 119.2, 119.0, 117.6, 117.4, 116.8, 114.6, 113.7, 113.4, 112.0, 110.6, 110.2, 109.8, 109.6, 59.9, 27.7, 27.5, 21.6. HRMS (ESI) calcd. for C_31_H_27_N_5_O_3_S (M+H)^+^ 550.1913, found 550.1908.

(S)-N’-(2,2-dimethylpropylidene)-5-phenyl-3-tosyl-1,2,3,6-tetrahydroazepino [4,5-b]indole-2-carbohydrazide (**6z**):

Yellow solid 0.41 g, yield 89%, mp 137–139 °C. ^1^H NMR (400 MHz, DMSO-d_6_) δ 10.92 and 10.65 (s, 1H), 10.26 and 10.14 (s, 1H), 7.76−6.83 (m, 15H), 5.84 and 5.33 (s, 1H), 4.04 and 3.86 (dd, J = 15.6, 4.4 Hz, 1H), 2.54 and 2.41 (s, 1H), 2.38 (s, 3H), 1.20 and 0.92 (s, 9H). ^13^C NMR (100 MHz, DMSO-d_6_) δ 167.6, 163.3, 159.1, 154.8, 144.8, 140.0, 139.6, 136.4, 135.5, 135.3, 132.8, 131.9, 130.6, 129.4, 129.2, 128.1, 128.0, 127.8, 127.5, 127.4, 125.2, 124.6, 121.6, 121.4, 119.1, 119.0, 117.6, 117.6, 116.6, 115.2, 112.1, 110.5, 109.9, 59.8, 34.9, 27.6, 27.4, 26.7, 21.5. HRMS (ESI) calcd. for C_31_H_32_N_4_O_3_S (M+H)^+^ 541.2273, found 541.2274.

(S)-N’-octylidene-5-phenyl-3-tosyl-1,2,3,6-tetrahydroazepino [4,5-b]indole-2-carbohydrazide (**6aa**):

Yellow solid, yield 54%, mp 87–88 °C. ^1^H NMR (400 MHz, DMSO-d_6_) δ 10.85 and 10.66 (s, 1H), 10.26 and 10.14 (s, 1H), 7.78−6.84 (m, 15H), 5.84 and 5.31 (d, J = 2.8 Hz, 1H), 4.00 and 3.81 (dd, J = 15.6, 5.6 Hz, 1H), 2.57 and 2.33 (dd, J = 15.2, 2.0 Hz, 1H), 2.37 (s, 3H), 2.02 and 1.58 (tq, J = 14.8, 7.2 Hz, 2H), 1.47−1.11 (m, 10H), 0.94−0.77 (m, 3H). ^13^C NMR (100 MHz, DMSO-d_6_) δ 166.9, 162.6, 151.7, 147.9, 144.4, 144.3, 139.6, 139.2, 136.0, 135.9, 135.1, 134.7, 132.3, 131.4, 130.2, 130.1, 129.0, 128.7, 127.6, 127.5, 127.4, 127.3, 127.0, 126.9, 124.8, 124.1, 121.2, 120.9, 118.6, 118.5, 117.0, 116.9, 116.2, 114.6, 111.6, 109.9, 109.3, 59.1, 54.7, 31.7, 31.6, 31.3, 31.1, 28.7, 28.6, 28.5, 28.4, 26.3, 25.8, 22.2, 22.1, 22.0, 21.0, 14.0, 13.9. HRMS (ESI) calcd. for C_34_H_38_N_4_O_3_S (M+H)^+^ 583.2736, found 583.2743.

### 3.3. Biological Assay

The anti-TMV, larvicidal, and fungicidal activities of the synthesized compounds were tested using our previously reported methods [28,29,30], the detail bioassay procedures are depicted in the Supplemetary Materials, Section S2: Detailed bioassay procedures for anti-TMV activities; Section S3: Stomach toxicity against Plutella xylostella; Section S4: Detailed bioassay procedures for fungicidal activities.

## 4. Conclusions

In summary, we designed and synthesized a series of novel derivatives containing azepino [4,5-b] indole and acylhydrazone moieties, and first evaluated their biological activities. Most of the compounds showed good to excellent anti-TMV activity compared to commercial ribavirin, among which, compounds **5c**, **6a**, **6h**, **6t**, **6v**, and **6y** displayed excellent anti-TMV activity in vivo. Meanwhile, we were pleased to find that almost all these derivatives showed good larvicidal activity against Plutella xylostella and these derivatives also showed a broad spectrum of fungicidal activity. This systematic study provides strong evidence that the rationality of our speculation and design ideology were preliminarily successful. Further studies on structural optimization are in progress in our laboratory.

## Figures and Tables

**Table 1:** In vivo anti-TMV activity of synthesized compounds **5a**−**5c**, **6a**−**6aa** ^1^.

Compd.	Concn. (µg/mL)	Inhibition Rate (%)		
		Inactivation Effect (%)	Curative Effect (%)	Protection Effect (%)
**5a**	500	44 ± 3	36 ± 2	42 ± 1
	100	10 ± 1	4 ± 1	14 ± 1
**5b**	500	50 ± 2	46 ± 4	43 ± 3
	100	20 ± 2	16 ± 1	19 ± 1
**5c**	500	55 ± 2	49 ± 3	50 ± 3
	100	22 ± 1	20 ± 1	26 ± 1
**6a**	500	52 ± 1	54 ± 3	47 ± 2
	100	16 ± 2	23 ± 1	18 ± 2
**6b**	500	35 ± 2		
**6c**	500	33 ± 1		
**6d**	500	41 ± 2	36 ± 2	38 ± 2
	100	16 ± 1	5 ± 2	8 ± 1
**6e**	500	46 ± 2	47 ± 3	37 ± 2
	100	10 ± 1	16 ± 1	12 ± 3
**6f**	500	40 ± 2	35 ± 2	44 ± 1
	100	15 ± 1	6 ± 1	10 ± 1
**6g**	500	49 ± 1	43 ± 4	50 ± 3
	100	21 ± 1	14 ± 1	17 ± 1
**6h**	500	54 ± 1	48 ± 2	45 ± 4
	100	23 ± 1	10 ± 4	20 ± 1
**6i**	500	40 ± 3		
**6j**	500	36 ± 1		
**6k**	500	42 ± 3	45 ± 2	46 ± 1
	100	9 ± 1	15 ± 1	12 ± 1
**6l**	500	34 ± 1		
**6m**	500	47 ± 2	49 ± 2	41 ± 3
	100	14 ± 1	9 ± 3	13 ± 1
**6n**	500	51 ± 4	42 ± 1	38 ± 3
	100	8 ± 2	11 ± 1	17 ± 1
**6o**	500	44 ± 1	43 ± 2	39 ± 1
	100	7 ± 4	12 ± 1	6 ± 1
**6p**	500	37 ± 1		
**6q**	500	38 ± 5		
**6r**	500	32 ± 1		
**6s**	500	40 ± 1	39 ± 1	43 ± 4
	100	11 ± 1	13 ± 1	8 ± 1
**6t**	500	52 ± 2	50 ± 2	46 ± 1
	100	18 ± 1	19 ± 1	11 ± 1
**6u**	500	39 ± 3		
**6v**	500	56 ± 2	46 ± 4	49 ± 3
	100	22 ± 1	19 ± 2	15 ± 1
**6w**	500	35 ± 2		
**6x**	500	38 ± 3		
**6y**	500	52 ± 3	54 ± 1	48 ± 1
	100	21 ± 1	24 ± 1	16 ± 1
**6z**	500	37 ± 3		
**6aa**	500	37 ± 3		
**ningnanmycin**	500	57 ± 2	55 ± 3	58 ± 1
	100	28 ± 1	26 ± 1	27 ± 2
**r** **abvirin**	500	40 ± 1	37 ± 1	39 ± 2
	100	12 ± 1	11 ± 1	15 ± 1

^1^ When the inactivation effect of a compound was less than 40%, its protection and curative effects were not determined.

**Table 2:** Larvicidal activity of compounds **5a**−**5c**, **6a**−**6aa** against *Plutella xylostella*.

Compd	Larvicidal Activity at Various Concentrations (mg/L)					
	600	200	100	50	25	10
**5a**	90 ± 0	50 ± 0	0			
**5b**	100	100	60 ± 0	30 ± 0		
**5c**	100	100	76 ± 6	40 ± 0		
**6a**	50 ± 0					
**6b**	90 ± 0	60 ± 0	30 ± 0			
**6c**	40 ± 0					
**6d**	70 ± 0	50 ± 0				
**6e**	60 ± 0	40 ± 0				
**6f**	70 ± 0	40 ± 0				
**6g**	100	80 ± 0	40 ± 0			
**6h**	100	70 ± 0	40 ± 0			
**6i**	70 ± 0	30 ± 0				
**6j**	60 ± 0	30 ± 0				
**6k**	100	100	80 ± 0	60 ± 0	40 ± 0	
**6l**	100	70 ± 0	30 ± 10			
**6m**	100	100	80 ± 0	76 ± 6	40 ± 0	
**6n**	90 ± 0	50 ± 0				
**6o**	70 ± 0	30 ± 0				
**6p**	76 ± 6	30 ± 0				
**6q**	40 ± 0					
**6r**	0					
**6s**	70 ± 0	50 ± 0				
**6t**	50 ± 0					
**6u**	90 ± 0	70 ± 0	40 ± 0			
**6v**	100	100	50 ± 0			
**6w**	50 ± 0					
**6x**	76 ± 6	43 ± 6				
**6y**	100	70 ± 0	40 ± 0			
**6z**	100	100	86 ± 6	70 ± 0	56 ± 6	30 ± 0
**6aa**	60 ± 0	20 ± 0				

**Table 3:** LC_50_ value of **6z** against *Plutella xylostella*.

Compd.	y = ax + b	LC_50_ (mg/L)	Correlation Coefficient
**6z**	y = 1.5974x + 2.8799	21.2	0.9989

**Table 4:** Fungicidal activity of compounds **5a**−**5c**, **6a**−**6aa** against fourteen kinds of phytopathogens ^1^.

Compd.	Inhibition Rate (% at 50 mg/L)													
	A.S.	F.G.	P.I.	P.C.	S.S.	B.C.	R.S.	F.C.	C.H.	P.P.	R.C.	B.M.	C.O.	F.M.
**5a**	56 ± 1	33 ± 1	51 ± 1	60 ± 2	69 ± 1	31 ± 1	28 ± 1	59 ± 1	83 ± 1	42 ± 2	90 ± 1	51 ± 1	50 ± 1	84 ± 1
**5b**	58 ± 1	52 ± 2	51 ± 1	65 ± 1	66 ± 1	47 ± 1	35 ± 1	39 ± 1	41 ± 3	27 ± 1	52 ± 1	36 ± 2	50 ± 1	52 ± 1
**5c**	45 ± 2	45 ± 2	74 ± 2	75 ± 2	76 ± 2	49 ± 2	66 ± 2	40 ± 2	52 ± 2	31 ± 2	60 ± 2	39 ± 1	47 ± 1	56 ± 1
**6a**	33 ± 1	32 ± 1	59 ± 1	40 ± 0	42 ± 1	38 ± 1	33 ± 1	33 ± 1	46 ± 1	20 ± 1	44 ± 1	23 ± 1	40 ± 1	66 ± 2
**6b**	47 ± 1	58 ± 1	39 ± 1	31 ± 1	57 ± 1	38 ± 1	28 ± 1	36 ± 1	56 ± 1	22 ± 1	53 ± 1	50 ± 1	53 ± 1	65 ± 1
**6c**	33 ± 1	56 ± 1	35 ± 1	35 ± 1	42 ± 1	25 ± 1	32 ± 1	41 ± 1	36 ± 1	20 ± 0	50 ± 1	33 ± 1	57 ± 1	61 ± 1
**6d**	52 ± 1	45 ± 1	43 ± 1	40 ± 1	49 ± 1	29 ± 1	37 ± 1	43 ± 1	62 ± 1	44 ± 1	92 ± 1	36 ± 1	47 ± 1	70 ± 0
**6e**	58 ± 1	44 ± 1	35 ± 1	50 ± 0	32 ± 1	34 ± 1	35 ± 1	39 ± 1	36 ± 1	20 ± 1	35 ± 1	61 ± 1	47 ± 1	66 ± 1
**6f**	50 ± 0	45 ± 1	30 ± 0	32 ± 1	42 ± 1	38 ± 1	39 ± 1	30 ± 0	52 ± 1	20 ± 1	57 ± 1	29 ± 1	50 ± 0	52 ± 1
**6g**	43 ± 1	47 ± 3	28 ± 1	35 ± 1	55 ± 1	29 ± 1	35 ± 1	36 ± 1	36 ± 1	20 ± 1	53 ± 1	36 ± 1	57 ± 1	56 ± 1
**6h**	33 ± 1	26 ± 1	43 ± 1	37 ± 1	32 ± 1	38 ± 1	28 ± 1	23 ± 1	42 ± 1	37 ± 1	64 ± 1	63 ± 1	50 ± 1	61 ± 1
**6i**	39 ± 1	34 ± 1	31 ± 1	30 ± 0	27 ± 2	41 ± 1	32 ± 1	36 ± 1	46 ± 1	30 ± 1	70 ± 0	26 ± 1	40 ± 0	59 ± 1
**6j**	45 ± 1	26 ± 2	35 ± 1	31 ± 1	42 ± 1	38 ± 1	30 ± 0	33 ± 1	46 ± 1	20 ± 1	53 ± 1	23 ± 1	50 ± 1	66 ± 1
**6k**	47 ± 2	26 ± 1	28 ± 1	30 ± 1	54 ± 1	38 ± 1	28 ± 1	35 ± 1	73 ± 2	27 ± 1	53 ± 1	36 ± 1	50 ± 0	70 ± 1
**6l**	59 ± 1	30 ± 1	37 ± 1	35 ± 1	30 ± 1	35 ± 1	31 ± 1	36 ± 1	78 ± 1	20 ± 1	46 ± 2	29 ± 1	40 ± 1	67 ± 1
**6m**	33 ± 1	34 ± 1	35 ± 1	36 ± 1	37 ± 1	29 ± 1	39 ± 1	52 ± 1	46 ± 1	44 ± 1	100	42 ± 1	50 ± 1	71 ± 1
**6n**	45 ± 1	26 ± 1	31 ± 1	40 ± 1	35 ± 1	34 ± 2	42 ± 1	36 ± 1	49 ± 1	37 ± 1	57 ± 1	36 ± 1	57 ± 1	61 ± 1
**6o**	54 ± 1	24 ± 1	35 ± 2	30 ± 1	36 ± 1	34 ± 1	28 ± 1	39 ± 1	46 ± 1	57 ± 1	59 ± 1	43 ± 1	60 ± 0	56 ± 1
**6p**	58 ± 3	33 ± 1	28 ± 1	25 ± 1	32 ± 1	31 ± 1	25 ± 1	43 ± 1	67 ± 3	59 ± 1	55 ± 1	45 ± 1	47 ± 1	66 ± 1
**6q**	53 ± 1	38 ± 1	38 ± 1	35 ± 1	40 ± 0	29 ± 1	51 ± 1	30 ± 0	36 ± 1	32 ± 1	57 ± 1	33 ± 1	57 ± 1	47 ± 1
**6r**	72 ± 1	45 ± 2	74 ± 2	24 ± 1	91 ± 1	38 ± 1	70 ± 0	30 ± 0	67 ± 1	20 ± 1	66 ± 1	57 ± 1	53 ± 2	56 ± 1
**6s**	70 ± 0	35 ± 1	43 ± 1	37 ± 1	27 ± 1	34 ± 1	34 ± 1	36 ± 1	57 ± 1	25 ± 1	50 ± 0	29 ± 1	60 ± 1	56 ± 1
**6t**	45 ± 1	26 ± 1	41 ± 1	30 ± 1	42 ± 1	29 ± 1	28 ± 2	46 ± 1	94 ± 1	27 ± 1	98 ± 1	33 ± 1	57 ± 1	100
**6u**	33 ± 1	23 ± 1	51 ± 1	40 ± 1	57 ± 2	59 ± 2	39 ± 1	36 ± 1	41 ± 1	25 ± 1	59 ± 1	26 ± 1	53 ± 1	56 ± 3
**6v**	70 ± 0	70 ± 0	35 ± 1	37 ± 1	49 ± 3	29 ± 1	35 ± 1	36 ± 1	57 ± 1	27 ± 1	55 ± 1	33 ± 1	57 ± 1	66 ± 1
**6w**	45 ± 1	59 ± 1	51 ± 1	42 ± 1	37 ± 1	36 ± 1	28 ± 1	36 ± 1	41 ± 1	20 ± 1	90 ± 0	36 ± 1	53 ± 1	56 ± 1
**6x**	58 ± 1	45 ± 1	46 ± 1	40 ± 1	44 ± 1	29 ± 1	25 ± 1	35 ± 1	52 ± 1	32 ± 1	53 ± 1	28 ± 1	67 ± 1	52 ± 1
**6y**	54 ± 1	58 ± 1	74 ± 1	50 ± 1	69 ± 1	56 ± 1	32 ± 1	36 ± 1	31 ± 1	30 ± 0	55 ± 1	39 ± 1	57 ± 1	61 ± 1
**6z**	58 ± 1	58 ± 1	45 ± 1	45 ± 2	69 ± 1	56 ± 1	40 ± 1	41 ± 1	73 ± 1	20 ± 1	63 ± 1	41 ± 1	60 ± 1	56 ± 1
**6aa**	50 ± 0	33 ± 1	66 ± 1	43 ± 1	86 ± 1	75 ± 1	35 ± 1	42 ± 1	46 ± 1	37 ± 1	63 ± 1	29 ± 1	61 ± 1	61 ± 1
chlorothalonil	38 ± 1	100	85 ± 1	90 ± 0	98 ± 1	82 ± 1	92 ± 1	71 ± 1	53 ± 1	10 ± 0	98 ± 1	56 ± 1	80 ± 1	41 ± 1

^1^ A.S., Alternaria solani; F.G., Fusarium graminearum; P.I., Phytophthora infestans; P.C., Phytophthora capsici; S.S., Sclerotinia sclerotiorum; B.C., Botrytis cinerea; R.S., Rhizoctonia solani; F.C., Fusarium oxysporum sp. cucumeris; C.H., Cercospora arachidicola Hori; P.P., Physalospora piricola; R.C., Rhizoctonia cerealis; B.M., Bipolaris maydis; C.O., Colletotrichum orbiculare; F.M., Fusarium moniliforme.

## Data Availability

Not applicable.

## Supplementary Materials

The following supporting information can be downloaded at https://www.mdpi.com/article/10.3390/molecules27196700/s1, Section S1: Copies of NMR spectra (Figures S1–S76); Section S2: Detailed bioassay procedures for anti-TMV activities; Section S3: Stomach toxicity against Plutella xylostella; Section S4: Detailed bioassay procedures for fungicidal activities.

## Sample Availability

Samples of the compounds are not available from the authors.
